# Lessons From Lighthouse: Operationalizing Technology to Support Older Adults in Affordable Housing Communities

**DOI:** 10.1093/geroni/igab046.1800

**Published:** 2021-12-17

**Authors:** David Lindeman

**Affiliations:** UC Berkeley, Oakland, California, United States

## Abstract

Lighthouse for Older Adults, an innovative public-private partnership, was developed in response to COVID-19 as a means of advancing telehealth for low-income older adults living in affordable housing communities. Residents of these communities often don’t have reliable access to devices, sufficient bandwidth for telehealth, or adequate social services, further complicated by the need for multi-lingual and culturally sensitive programs. This presentation will share program implementation strategies and outcomes, including the essential role telehealth services play in the care and wellbeing of older adults during and beyond COVID-19. This session will review evidence-based components of a telehealth intervention, including digital literacy training and technology support. Key drivers for successful implementation (e.g., peer led training, user input into technology selection) as well as barriers to implementation (e.g., broad band installation, internet service availability/cost, tech support) will be reviewed. Lessons learned through program replication and scaling of Lighthouse telehealth services will be discussed.

